# Effect of organic matter on estuarine flocculation: a laboratory study using montmorillonite, humic acid, xanthan gum, guar gum and natural estuarine flocs

**DOI:** 10.1186/1467-4866-15-1

**Published:** 2014-01-03

**Authors:** Yoko Furukawa, Allen H Reed, Guoping Zhang

**Affiliations:** 1 Naval Research Laboratory, Seafloor Sciences Branch, Stennis Space Center, Mississippi 39529, USA; 2 Department of Civil and Environmental Engineering, University of Massachusetts Amherst, Amherst, Massachusetts 01003, USA

**Keywords:** Flocculation, Aggregation, Colloid, Montmorillonite, Humic, Biopolymer, Organic matter, Estuary, River

## Abstract

**Background:**

Riverine particles undergo a rapid transformation when they reach estuaries. The rapid succession of hydrodynamic and biogeochemical regimes forces the particles to flocculate, settle and enter the sediment pool. The rates and magnitudes of flocculation depend on the nature of the particles which are primarily affected by the types and quantities of organic matter (OM). Meanwhile, the OM characteristics vary widely between environments, as well as within a single environment due to seasonal climate and land use variability. We investigated the effect of the OM types and quantities through laboratory experiments using natural estuarine particles from the Mississippi Sound and Atchafalaya Bay as well as model mixtures of montmorillonite and organic molecules (i.e., biopolymers (guar/xanthan gums) and humic acid).

**Results:**

Biopolymers promote flocculation but the magnitude depends on the types and quantities. Nonionic guar gum yields much larger flocs than anionic xanthan gum, while both of them exhibit a nonlinear behavior in which the flocculation is the most pronounced at the intermediate OM loading. Moreover, the effect of guar gum is independent of salinity whereas the effect of xanthan gum is pronounced at higher salinity. Meanwhile, humic acid does not affect flocculation at all salinity values tested in this study. These results are echoed in the laboratory manipulation of the natural estuarine particles. Flocculation of the humic acid-rich Mississippi Sound particles is unaffected by the OM, whereas that of biopolymer-rich Atchafalaya Bay particles is enhanced by the OM.

**Conclusions:**

Flocculation is positively influenced by the presence of biopolymers that are produced as the result of marine primary production. Meanwhile, humic acid, which is abundant in the rivers that drain the agricultural soils of Southeastern United States, has little influence on flocculation. Thus, it is expected that humic acid-poor riverine particles (e.g., Mississippi River, and Atchafalaya River, to a lesser degree) may be prone to rapid flocculation and settling in the immediate vicinity of the river mouths when mixed with biopolymer-rich coastal waters. It is also expected that humic acid-rich riverine particles (e.g., Pearl River) may resist immediate flocculation and be transported further away from the river mouth.

## Background

When riverine suspended particles are transported downstream and enter the land-ocean interface and estuaries, they undergo a rapid transformation including flocculation and settling. The past 30 years of field observations have found that the rapid succession of hydrodynamic and biogeochemical regimes in the vicinity of the land-ocean interface cause the transformation [1,2]. The rate and magnitude of this transformation are important. If the transformation is rapid, then the riverine particles will flocculate, settle and enter the bottom sediment pool within the immediate vicinity of the river mouth. On the other hand, if the transformation is slow, the riverine particles will remain suspended and be transported further away from the source rivers and onto the continental shelf and beyond.

However, a generalized, robust and comprehensive understanding of this transformation has not fully been achieved. This is primarily because the net effect (for example, net rates of floc growth and settling) depends on the nature of the particles, especially on the types and quantities of organic matter (OM), which varies widely between different rivers as well as within a single river due to seasonal climate and land use variability. Previous flocculation studies have either focused on: (i) the effect of salinity and resulting variation in the surface chemistry of the suspended particles [3,4], (ii) the effect of single species of organic matter [5,6]; or (iii) the in-depth investigations of a single depositional environment (e.g., Mississippi Delta) [7]. As a result, systematic assessments between different types of OM species and between different depositional environments have not been conducted.

Consequently, the purpose of this study was to evaluate the effect of different types of OM species (i.e., anionic biopolymer, nonionic biopolymer and humic substances) on the estuarine and coastal flocculation of suspended particles. The evaluation employed a series of laboratory experiments in which an aqueous model suspended mineral (powdered montmorillonite) was mixed with different types of OM species (xanthan gum, guar gum and humic acid) with a range of OM-to-clay loading ratios, and then mixed with artificial seawater to yield a range of salinity values. Further, the evaluation also took advantage of natural estuarine samples from two environments, one from the marine biopolymer-rich Atchafalaya Bay area, and the other from humic acid-rich Mississippi Sound. The different flocculation behaviors of samples from these two environments underscore the laboratory findings in which the effect of OM is diverse and nonlinear depending on the OM species.

## Methods

### Experiments with simulated riverine particles

#### Starting materials

This study used montmorillonite, a clay mineral abundant in many estuarine waters and sediments such as the Mississippi River Delta sediments [8], to simulate the clay mineral fraction of the suspended particles in rivers and estuaries. Montmorillonite powder (Ward’s Scientific, powdered Na-bentonite, 46E0435) was used without further size fractionation or purification. The median diameter of the dispersed montmorillonite powder in water was determined by the laser diffraction technique (see below) to be 3.5 μm.

Humic acid (HA) is ubiquitous and abundant in many river and estuarine waters, including those in the vicinity of the Mississippi Sound [9]. The HA sample (Aldrich, humic acid sodium salt, 60% humic acid, CAS 68131-04-4) was dissolved in milli-Q water to yield 100 mg L^-1^ stock suspension. Xanthan gum (Spectrum Chemical, CAS 11138-66-2), an anionic hydrophilic biopolymer often used as a proxy for microbial extracellular polymeric substances (EPS) in laboratory experiments [10], was also dissolved in milli-Q water to yield 100 mg L^-1^ stock suspension. Guar gum (Fisher Scientific, CAS 9000-30-0), a non-ionic hydrophilic biopolymer, was combined with milli-Q water and continuously stirred overnight to yield 100 mg L^-1^ stock suspension. All OM stock suspensions were filtered through 0.45 μm-pore syringe filters prior to use, and used or discarded within 72 hours.

Artificial seawater (ASW) was prepared by dissolving 23.93 g NaCl, 4.01 g Na_2_SO_4_, 0.67 g KCl, 0.20 g NaHCO_3_, 10.83 g MgCl_2_ · 6H_2_O, and 1.52 g CaCl_2_ · 2H_2_O to 1 L milli-Q water (modified after [11]). The nominal salinity of ASW was 35 psu.

#### Experimental procedure

For each experimental run, 100 mg of the montmorillonite powder was combined with an appropriate mixture of milli-Q water and OM stock solution, as well as ASW, to yield a 400 mL aqueous mixture. The total suspended solid (TSS) in each experimental run was approximately 250 mg L^-1^ (dry weight basis). This TSS value was dictated by the particle size analyzer used in this study, as it was the optimal value for the analyzer’s laser optics. The value is high, yet within the same order of magnitude as the TSS values previously measured in Lower Mississippi River (i.e., <10 - 262 mg L^-1^, [12,13]) and in Pearl River (4 – 57 mg L^-1^[13]). The organic matter stock solutions were introduced such that the total organic carbon (TOC) concentrations varied between 0 and 20 mg L^-1^ (i.e., mixing ratio of 0 – 8 weight% TOC/TSS), and salinity varied between 0 and 17.5 psu. The experiments were aimed at simulating the estuarine mixing. Consequently, montmorillonite, milli-Q water and the OM stock solution were combined and stirred for at least 5 minutes before ASW was added. Once ASW was added, the floc size distribution of the suspended materials was measured every 5 – 10 minutes using the laser diffraction technique (see below) until the size distribution reached the steady state. The solution pH was monitored using a combination electrode calibrated with NBS buffers, and was adjusted to be circumneutral (i.e., between 6.9 and 7.1) using 0.1 N NaOH or 0.1 N HCl in order to minimize uncertainty due to small fluctuation in the pH values. At circumneutral pH, the surface properties of montmorillonite and OM molecules are relatively pH-insensitive. The steady-state median diameter (*d*_50_) is reported from each experimental run after the size distribution was determined by laser diffraction (see below) in this study.

### Natural estuarine particles

#### Field sampling

**Figure 1:**
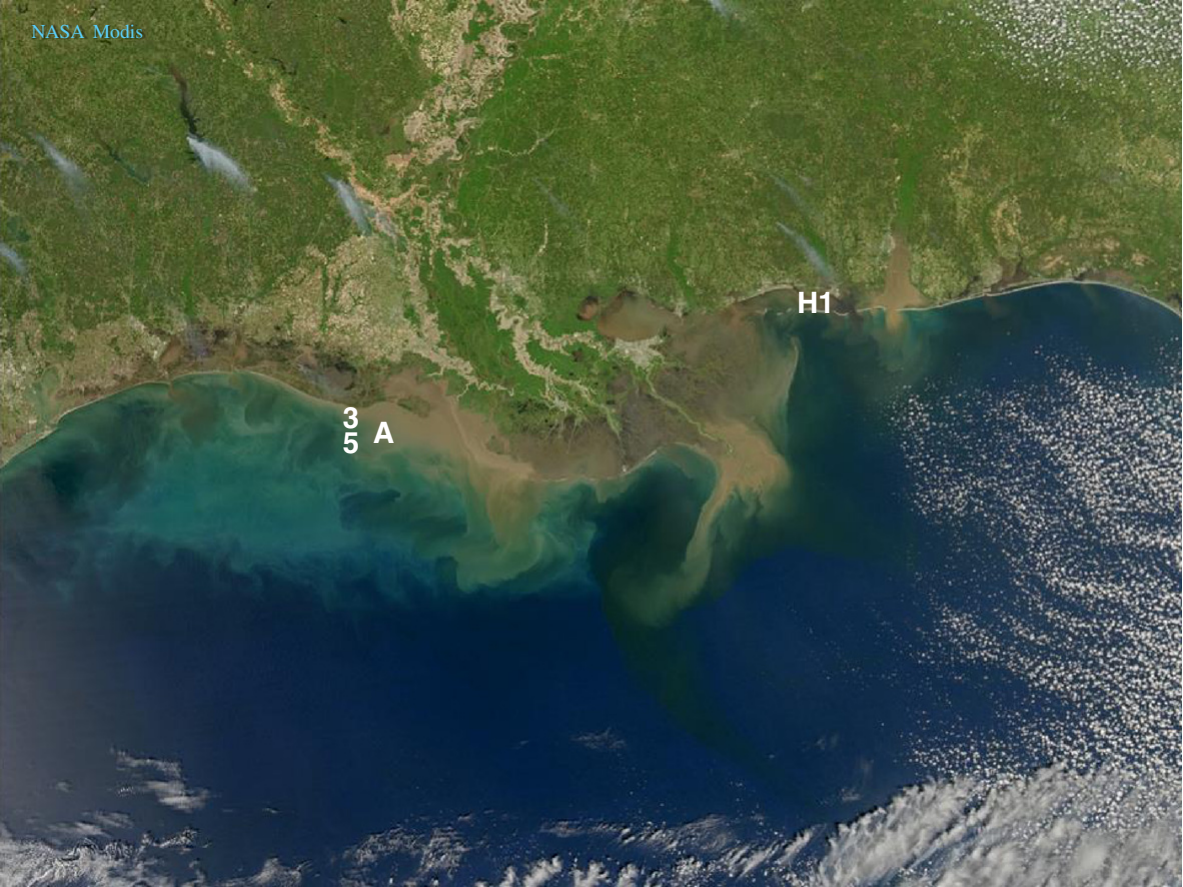
The samples of natural estuarine particle were collected in the field stations near Horn Island (Station H1) in the Mississippi Sound, and near Atchafalaya Bay off Louisiana coast (Stations A3 and A5) (Satellite image courtesy of NASA MODIS).

Natural estuarine particles were collected in the field stations near Horn Island in the Mississippi Sound (H1, +30° 15′ 59.76″, -88° 45′ 16.38″) and in the vicinity of Atchafalaya Bay (A3, +29° 28′ 48.18″, -92° 25′ 32.16″ and A5, +29° 18′ 0.00″, -92° 6′ 0.00″) (Figure 1). The environmental parameters of the stations at the time of sampling are summarized in Table 1. The Mississippi Sound station receives particulate matters from humic-rich Pearl, Jordan and Wolf Rivers that drain the agricultural soils of the state of Mississippi and Louisiana. The Atchafalaya Bay stations receive discharge from the Atchafalaya River, which is a distributary of the Mississippi River, draining the Atchafalaya Basin floodplains. The dissolved organic carbon (DOC) concentration of the Pearl River fluctuates between 500 and 1,900 μM throughout the year [14], whereas that of the Atchafalaya River is lower, fluctuating between 350 and 600 μM throughout the year [15].

**Table 1:** Environmental parameters of the field stations determined at the time of sampling

**Station**	**Sampling date**	**Water depth (m)**	**Water temperature (°C)**	**Salinity (psu)**	**Flow velocity (mm s**^**-1**^**)**
H1	Oct 25, 2012	3.8	16.0	25.0	129 ± 70
A3	Apr 3, 2013	3.4	19.1	19.4	818 ± 172
A5	Apr 4, 2013	7.3	19.2	30.1	713 ± 498

The suspended particulate materials were collected from immediately above the water-sediment interface by a diver (at H1) or by a Nepheloid layer sampler (at A3 and A5). The samples were kept in a refrigerator as hydrated slushes (the approximate porosity of 80% or greater) without drying until the time of experiments. The organic carbon to mineral weight ratio for the surface sediment samples from H1, A3 and A5 are 1.35%, 2.29% and 1.90% TOC, respectively.

#### Experimental procedure

The natural untreated estuarine particles, which contained intrinsic OM, were subjected to a range of salinity (0 – 17.5 psu) as they were suspended into the mixture of milli-Q water and ASW. The salinity was confirmed by analyzing the aqueous conductivity. The solution pH was monitored using a combination electrode calibrated with NBS buffers, and was adjusted to be between 6.9 and 7.1 using 0.1 N NaOH or 0.1 N HCl. Their steady-state median diameters at various salinity values are reported in this study. The total suspended solid concentration (equivalent dry weight) in each run was approximately 250 mg L^-1^. The steady-state median floc size for the natural surface sediment samples from H1, A3 and A5 at zero salinity were determined by laser diffraction (see below) to be 6.81 μm, 6.45 μm and 10.7 μm, respectively. It should be noted that the median size was determined from the entire population of the natural particles which include sand- and silt-sized particles as well as the clay-sized particles that some of which are flocculated while others are discrete individual particles.

The natural estuarine particles were also pretreated with 30% hydrogen peroxide in order to remove OM, by soaking 5 grams of slurry in 5 mL 30% H_2_O_2_ overnight. After the OM removal, the remaining OM-free particles were suspended in a mixture of milli-Q water and ASW (salinity ranging between 0 and 17.5 psu). The solution pH was monitored using a combination electrode calibrated with NBS buffers, and was adjusted to be between 6.9 and 7.1 using 0.1 N NaOH or 0.1 N HCl. The steady-state median diameters were analyzed. The median floc size for the OM-free surface sediment samples from H1, A3 and A5 at zero salinity were determined by laser diffraction (see below) to be 7.17 μm, 5.44 μm and 10.2 μm, respectively.

#### Size analysis by laser diffraction

The floc size distribution of the montmorillonite-OM and estuarine particle suspensions was characterized using the CILAS® 1190 laser diffraction-based particle size analyzer (PSA) (CILAS, Orléans, France) [16,17]. The PSA system is composed of: (i) the sample well, where the aqueous suspension is continuously stirred with a paddle in order to avoid settling of larger flocs and particles; (ii) the laser analysis chamber, where the aqueous suspension is subjected to two laser beams (Wavelength = 635 and 830 nm) for laser diffraction; and (iii) the closed-loop plumbing system with a peristaltic pump that continuously circulates the aqueous sample suspension between the well and chamber.

All particles diffract laser, and the diffraction angle is directly correlated to the particle diameter. The PSA system determines the size distribution of flocs by measuring the intensity of the laser as a function of the diffraction angle, and converting it to the spherical-equivalent size distribution [16,18]. This study uses the median diameter in terms of volume, *d*_50_. This means that in each system, flocs with the diameter value of *d*_50_ or less represent 50% of the total volume of the flocs.

It took a typical sample up to a few hours to reach the steady state size distribution. Whereas the laser beams were activated only intermittently (i.e., every ~5 minutes) for the time-series size measurements, the stir paddle and peristaltic pump stayed on for the entire duration of each run. We assume that the steady state size distribution is being achieved in the sample well rather than in the plumbing system or in the laser chamber, as the residence time in the well is far greater than that in the plumbing system or in the laser chamber. For all experiments, the stir paddle was operated at 210 rpm, and the peristaltic pump was operated at 60 rpm. The mean shear rate (*G* (s^-1^)) experienced by the sample flocs in this sample well can be estimated using:

$$G=\frac{52.3b_{d,p}A_{p}s^{3}R_{p}^{3}}{v_{\omega }V_{T}}$$

where *b*_*d,p*_ is a drag coefficient for the paddle (= 1.0 estimated), *A*_*p*_ is the cross sectional area of the paddle normal to the fluid (= 4.29 cm^2^ for our CILAS sample well), *s* is the paddle rotation velocity (a constant value of 210 rpm or 3.5 s^-1^ was used throughout the study), *R*_*p*_ is the paddle rotation radius (= 2.6 cm for our CILAS sample well), *υ*_*w*_ is the momentum dissipation constant (= 0.01 cm^2^ s^-1^), and *V*_*T*_ is the volume of the sample within the well (≅ 400 mL for our experiments) [19]. The resulting mean shear rate for our experiments is *G* = 202 s^-1^. This value is higher than the typical shear rate values encountered in most estuarine environments [20]. However, we maintained this shear rate throughout the experiments as a slower paddle speed *s* caused some flocs, especially those larger flocs formed in the montmorillonite – guar gum systems, to settle out and cause inaccurate size measurements.

## Results and discussion

### Experiments with simulated riverine particles

**Figure 2:**
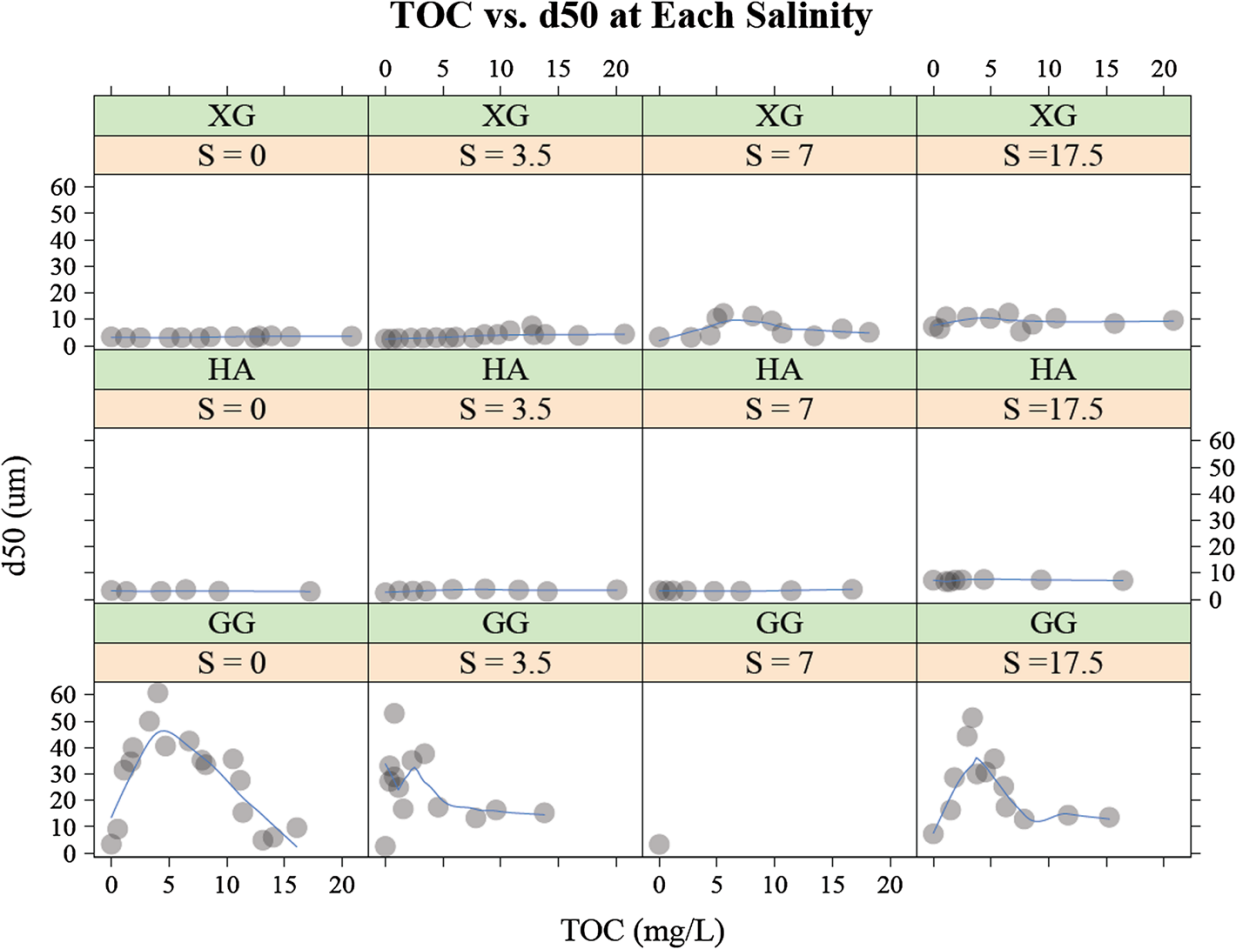
**The steady-state median floc size (*d***_**50 **_**μm) for the systems containing 250 mg L**^**-1 **^**montmorillonite and varying quantities (0 – 20 mg L**^**-1 **^**in terms of total organic carbon (TOC)) of organic matter (either xanthan gum (XG), humic acid (HA) or guar gum (GG)) was determined at four discrete salinity values (i.e., S = 0, 3.5, 7 and 17.5 psu).**

The effect of OM on estuarine flocculation is diverse and nonlinear. Among the OM types tested in this study, guar gum is the most effective in enhancing the flocculation (Figure 2). However, the effect is non-linear. The steady-state median floc size exhibits the maximum value at around 5 mg L^-1^ TOC loading. When the guar gum loading exceeds 5 mg L^-1^ TOC, the flocs are smaller. It should also be noted that the effect of salinity is negligible, meaning that the overall shape of the TOC vs. *d*_50_ plots are similar for the guar gum + montmorillonite systems at all different salinity values. The other two OM types (i.e., xanthan gum and humic acid) are not nearly as effective as guar gum in enhancing flocculation at all salinity values and OM loadings we tested.

**Figure 3:**
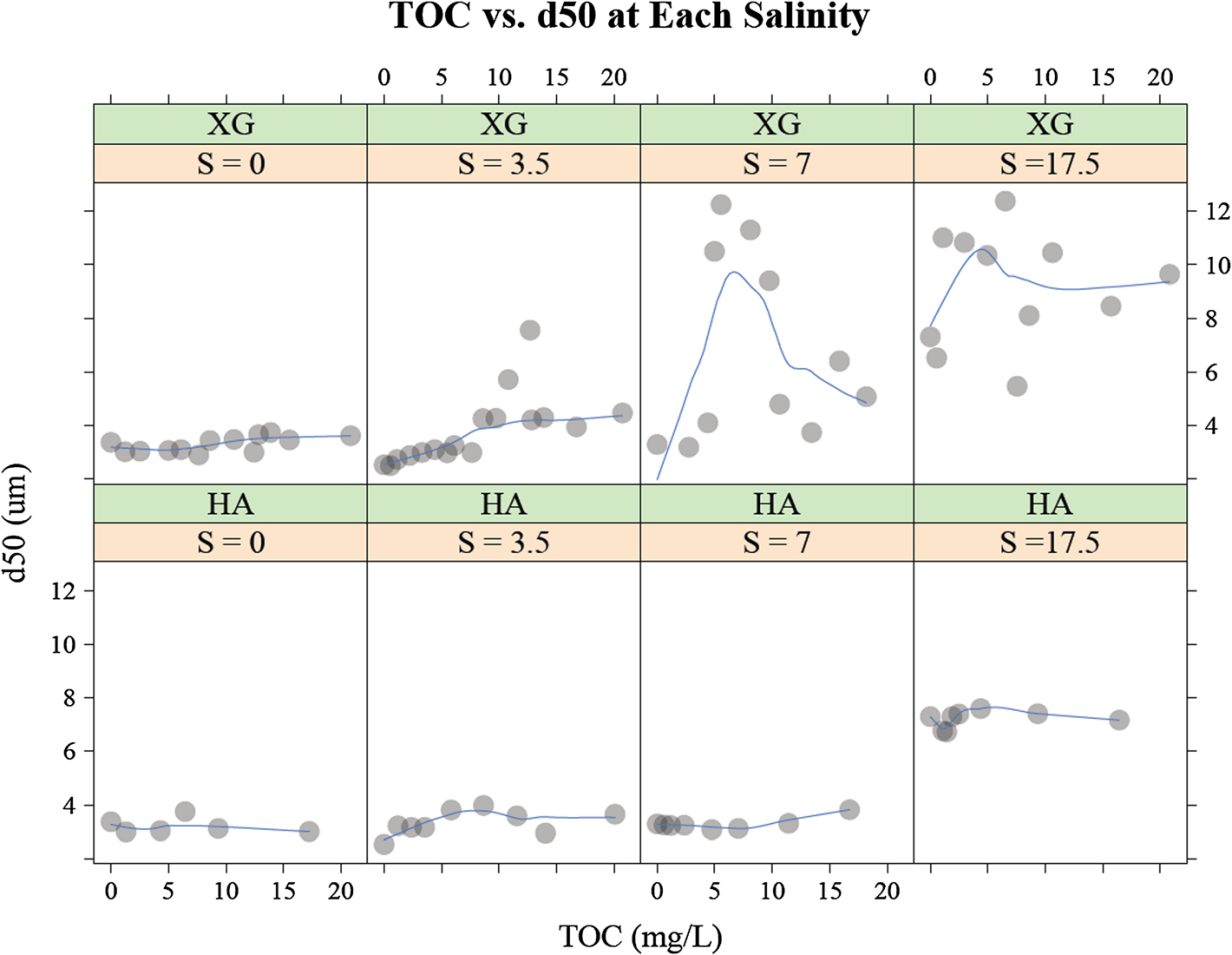
**The steady-state median floc size (*d***_**50 **_**μm) for the systems containing 250 mg L**^**-1 **^**montmorillonite and varying quantities (0 – 20 mg L**^**-1 **^**in terms of total organic carbon (TOC)) of organic matter (either xanthan gum (XG) or humic acid (HA)) was determined at four discrete salinity values (i.e., S = 0, 3.5, 7 and 17.5 psu).** This figure shows the same xanthan gum and humic acid data presented in Figure 2, but on a different y axis scale.

Whereas the magnitude is much smaller, xanthan gum, also a biopolymer, enhances flocculation (Figure 3). Besides the magnitude of the flocculation, xanthan gum differs from guar gum in that its flocculation is affected by salinity. The effect of xanthan gum is insignificant at salinity = 0 psu (i.e., the slope of the TOC vs. *d*_50_ plot is nearly zero), while the effect is more pronounced in higher salinity solutions (i.e., the slope of the TOC vs. *d*_50_ plot is nonzero). The non-linear nature of the effect is also apparent. At salinities of 7 and 17.5 psu, the greatest flocculation occurs when the xanthan gum loading is approximately 5 mg L^-1^ TOC. At higher TOC loading levels, the steady state floc sizes are smaller.

**Figure 4:**
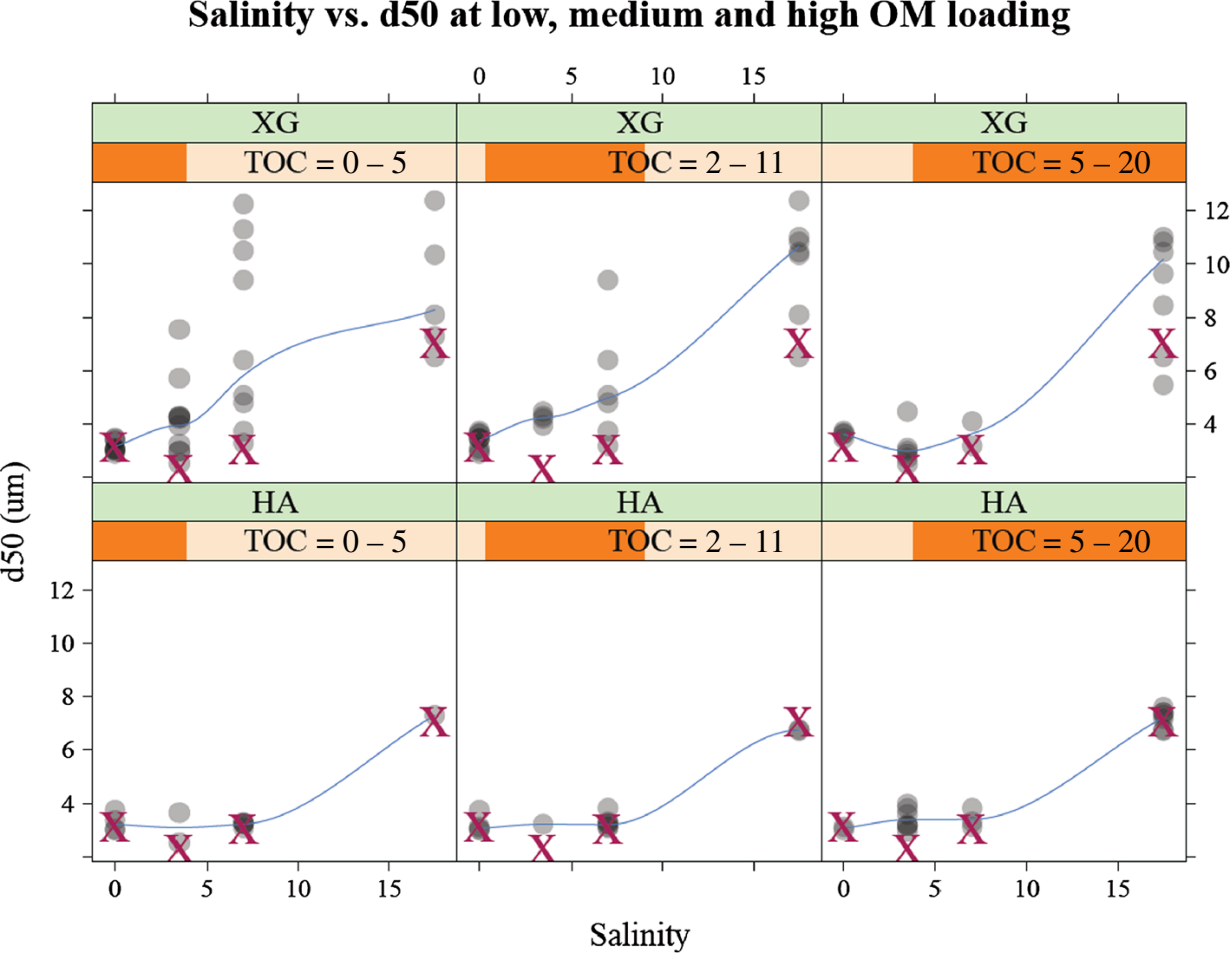
**The median floc diameter is shown as a function of salinity for three overlapping ranges of organic matter loading (i.e., TOC = 0 – 5, 2 – 11, and 5 – 20 mg L**^**1-**^**).** The data for the size of organic matter-free flocs are shown using red x’s for comparison.

Humic acid behaves differently from the biopolymers discussed above. Humic acid does not affect flocculation, as the humic acid vs. *d*_50_ plots in Figure 3 exhibit slopes that are nearly zero under every salinity level tested in this study. The ineffectiveness of humic acid may be better illustrated in Figure 4 in which the same data as Figure 3 are plotted in terms of salinity vs. median steady state floc diameter at three different TOC loading levels. The data for the median steady-state size of OM-free flocs are overlaid as red x’s for comparison. The xanthan gum graphs show that there is a significant difference between the OM-free plots (red x’s) and xanthan gum + montmorillonite plots (gray circles) at both medium and high xanthan gum loadings. On the other hand, there is virtually no difference between the OM-free plots (red x’s) and humic acid + montmorillonite plots (gray circles) regardless of the humic acid loading. This indicates that humic acid does not affect montmorillonite flocculation either negatively or positively at all salinity ranges tested.

In summary, three types of OM tested in this study behave very differently from one another with regard to their flocculation behavior in aqueous systems that contain montmorillonite and varying concentrations of ASW.

– Guar gum is a very effective flocculant when its loading is at around 5 mg L^-1^ TOC. When the loading exceeds this value, however, it becomes less effective as a flocculant. Another notable feature of guar gum is that its effectiveness does not change as a function of salinity.

– Xanthan gum is a moderately effective flocculant. Similar to guar gum, its effectiveness is peaked at around 5 mg L^-1^ TOC loading. On the other hand, unlike guar gum, xanthan gum is more effective as a flocculant at relatively higher salinities.

– Humic acid does not affect flocculation either positively or negatively regardless of salinity.

### Natural estuarine particles

**Figure 5:**
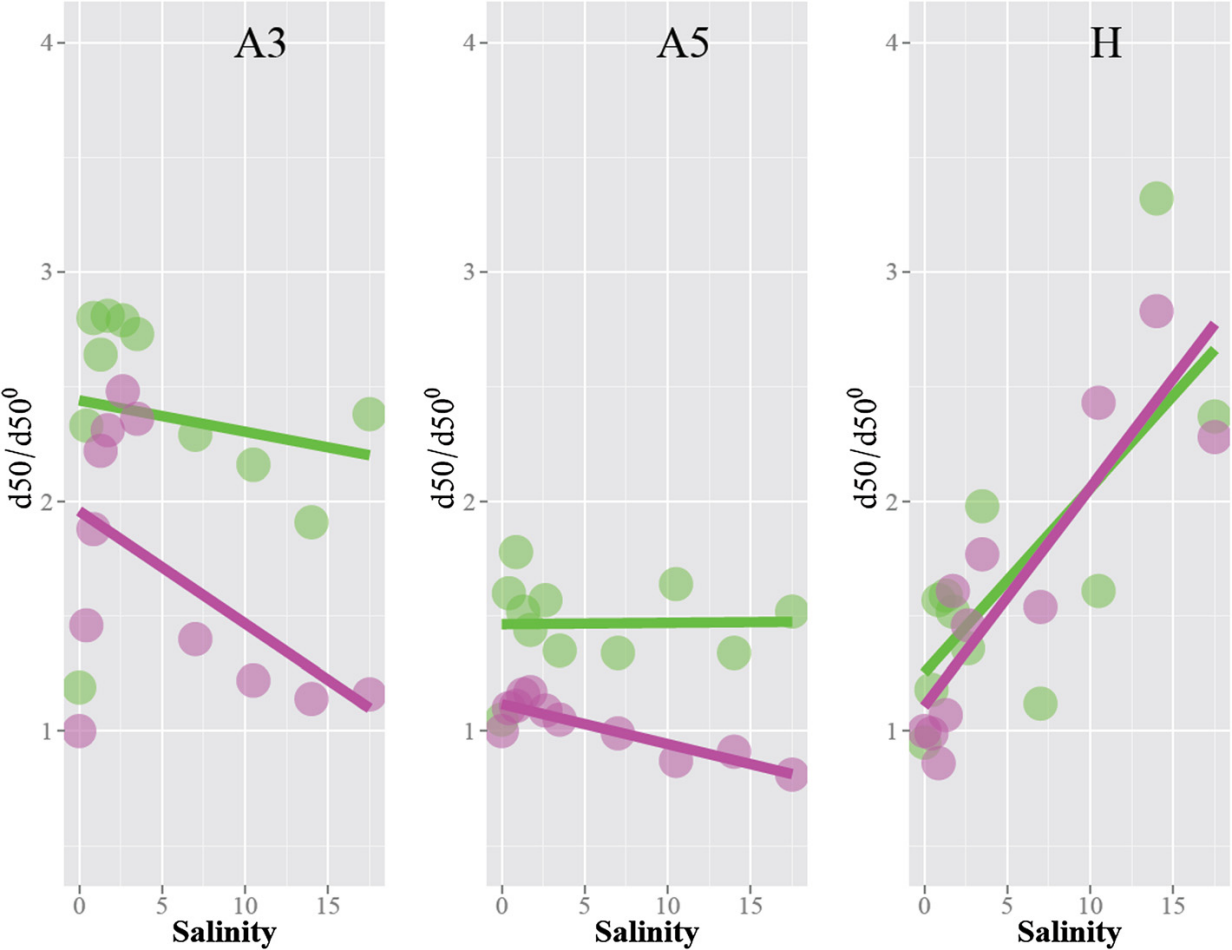
**The steady-state median floc size (*d***_**50 **_**μm) of untreated flocs (green circles) and H**_**2**_**O**_**2**_**-treated OM-free samples (pink circles) as a function of salinity.** The floc samples were collected near Atchafalaya Bay (A3 and A5) and in the Mississippi Sound near Horn Island (H1) before subjected to the laboratory experiments. The solid lines represent the linear fit to the data. The size is normalized for the OM-free steady-state median size at zero salinity. There is a clear separation between untreated and OM-free flocs for the Atchafalaya Bay samples while there is no discernible difference between untreated and OM-free floc sizes for the Horn Island sample.

The laboratory results above are analogous to what we observed for the natural estuarine particles (Figure 5). Atchafalaya Bay particles, which are poor in terrestrial riverine OM and naturally enriched with marine biopolymers presumably similar to guar gum and xanthan gum [14,21,22], exhibit the positive flocculation influence of the biopolymers. The steady-state median floc size of the untreated Atchafalaya flocs is significantly larger than that of the H_2_O_2_-treated OM-free particles at all salinity levels. Presumably, the slight salinity dependency implies that the biopolymers in the Atchafalaya Bay particles may be predominantly ionic biopolymer similar to xanthan gum. In reality, it is likely that the biopolymer in nature is a mixture of various biopolymers that include both anionic and nonionic biopolymers whose exact long-range interaction characteristics are highly dependent on a range of environmental factors [23]. Meanwhile, Mississippi Sound particles, which are naturally enriched with humic acid originated in the agricultural soils of the region [14,22], exhibit steady-state median floc sizes that are similar between the untreated and H_2_O_2_-treated samples.

### Conceptual models for estuarine flocculation

**Figure 6:**
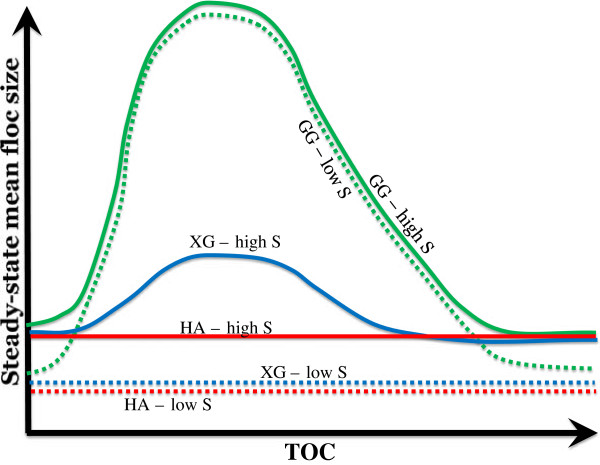
Schematic diagram showing the effect of various organic matter (GG: guar gum; XG xanthan gum; HA: humic acid) on the flocculation of aqueous montmorillonite suspension at low (~0 psu) and high (~7-17.5 psu) salinity.

The schematic diagram shown in Figure 6 summarizes that the effect of OM on montmorillonite flocculation is diverse and nonlinear. These observations may be illustrated by the following conceptual models:

(1) Humic acid exerts steric repulsion at a wide range of salinity values [24,25]. As a result, humic acid does not affect flocculation either at low or high salinity.

(2) The positive flocculation behavior of biopolymers is likely due to the polymer bridging [26].

(3) The nonlinear nature of the biopolymers as the flocculant may be due to the difference between the clay-biopolymer bond and biopolymer-biopolymer bond. Previous studies have confirmed that the excess polymer has a negative effect on polymer bridging [27] and thus results in the nonlinear flocculation behavior as a function of biopolymer loading. This can be conceptualized as the monolayer of polymers vs. multilayers of polymers. Monolayer, or single strands of polymers can act as bridges by adsorbing onto the montmorillonite surface at both ends of the polymer strands. The attachment between clay surfaces and polymer ends may be due to hydration pressure or non-charge transfer Lewis acid–base interactions [26]. Meanwhile, as homoflocculation between biopolymer molecules alone is unlikely because of the hydrophilicity [27], multiple layers of biopolymers on the clay surface due to excess biopolymers may lead to fewer bridges and less flocculation.

(4) Salinity influences the xanthan gum flocculation but not the guar gum flocculation. Ionic biopolymers (e.g., xanthan gum) become less polar at higher salinities due to specific adsorption of cations. The reduction in polarity can lead to the reduction in repulsive hydration [28,29] as well as the reduction in electrostatic repulsion [30]. Consequently, more polymer bridging would form. For nonionic biopolymers (e.g., guar gum), a change in salinity affects neither the polarity and the resulting repulsive hydration, nor the electrostatic repulsion.

## Conclusions

Different types of estuarine organic matters affect the estuarine sediment flocculation in different fashions. Guar gum, a nonionic biopolymer, significantly enhances flocculation. However, the flocculation effect is nonlinear, meaning that the relationship between guar gum loading and steady-state median floc size exhibit distinct maxima at around 5 mg L^-1^ TOC loading. The effect of guar gum on estuarine flocculation appears to be independent of salinity. Xanthan gum, an anionic biopolymer, also enhances flocculation, although the magnitude of enhancement is less than that of guar gum. The effect of xanthan gum is also nonlinear with the maximum flocculation observed at around 5 mg L^-1^ TOC loading. Xanthan gum is different from guar gum in that the effect of xanthan gum on estuarine flocculation is positively correlated with salinity. Atchafalaya Bay particles, which contain abundant marine biopolymers, exhibit enhanced flocculation due to the presence of biopolymers. The Atchafalaya Bay particle flocculation is positively correlated to salinity, suggesting that the biopolymer in the vicinity of Atchafalaya Bay is ionic.

Humic acid does not affect estuarine flocculation either positively or negatively, regardless of the salinity. The Mississippi Sound particles, which contain abundant humic acid originated in the agricultural soils of the source regions, exhibit no change in the flocculation due to humic acid. Humic acid’s tendency to inhibit flocculation and settling may promote the Mississippi Sound particles to remain in the water column to be transported from the estuarine environments into the continental shelf, while the marine biopolymer-rich Atchafalaya Bay particles may flocculate and rapidly settle to the bottom in the immediate vicinity of the Bay, although the seasonal variation in the biopolymer loading (i.e., spring bloom) may overload the system to inhibit flocculation.

This study underscores the importance of the types and quantities of organic matter in estuarine flocculation and sediment dynamics. The effect of organic matter on flocculation is diverse and nonlinear. The site-specific and seasonally variable characteristics of the types and concentrations of organic matter need to be taken into consideration when the transport dynamics of estuarine particles is investigated.

## Competing interests

The authors declare that they have no competing interests.

## Authors’ contributions

YF designed the experiments and conducted the size analysis. AR selected the field stations and carried out the sample collection. GZ advised YF in the experimental design and participated in the manuscript draft. All authors read and approved the final manuscript.
